# Interfaith Spiritual Care: A Systematic Review

**DOI:** 10.1007/s10943-017-0369-1

**Published:** 2017-02-15

**Authors:** Anke I. Liefbroer, Erik Olsman, R. Ruard Ganzevoort, Faridi S. van Etten-Jamaludin

**Affiliations:** 1 Faculty of Theology, VU Amsterdam, De Boelelaan 1105, 1081 HV Amsterdam, The Netherlands; 2 Department of General Practice, Section of Medical Ethics, Academic Medical Center, University of Amsterdam, Amsterdam, The Netherlands; 3 Department of Neurology, Section of Ethics and Law, Leiden University Medical Center, Leiden, The Netherlands; 4 Department of Spiritual Care, Hospice Bardo, Hoofddorp, The Netherlands; 5 Medical Library, Academic Medical Center, University of Amsterdam, Amsterdam, The Netherlands

**Keywords:** Interfaith, Spirituality, Religion, Caregivers, Patients, Chaplaincy, Review

## Abstract

Although knowledge on spiritual care provision in an interfaith context is essential for addressing the diversity of patients’ religious and spiritual needs, an overview of the literature is lacking. Therefore, this article reviews the empirical literature on interfaith spiritual care (ISC) in professional caring relationships. A systematic search in electronic databases was conducted to identify empirical studies published after 2000. Twenty-two studies were included. The quality of the included studies was assessed, and their results were thematically analyzed. The majority were conducted in North America, mainly using qualitative methods and focusing on professional caregivers, who had a variety of professional and spiritual backgrounds. Two core categories were identified: (1) normativity: reasons for (not) wanting to provide ISC, in which universalist and particularist approaches were identified; and (2) capacity: reasons for (not) being able to provide ISC, which included the competences that health care professionals may need when providing ISC, as well as contextual possibilities and restraints. This systematic review identifies gaps in the literature and indicates that future studies have to explore patient perspectives on ISC.

## Introduction

Over the past decades, the religious and spiritual landscape in Western societies has been transforming rapidly because of processes such as subjectivization, individualization, secularization, globalization, and pluralization (Woodhead et al. 2016). These changes are relevant for the field of spiritual care because they lead to a diversity of spiritual, religious, and cultural needs, which requires professional caregivers to deal with these diverse needs.

Several authors have noted the significance of addressing patients’ diverse needs in health care settings (as well as in the military and in penitentiary institutions). Some have provided practical guidelines and recommendations for health care professionals, other than chaplains, on spiritual care for patients of diverse religious traditions (Miklancie 2007; Richards and Bergin 2014; Walsh 2010), and others have plead for an “inter-” or “multifaith” model of spiritual care for spiritual care providers or chaplains (Gatrad et al. 2003, 2004; Schipani and Bueckert 2009). In these discussions, the distinction is often made between an “interfaith,” “generic,” or “multifaith” approach and a “faith-specific” approach. In the first approach, chaplains are trained to provide spiritual care to patients of all faiths; in the second, chaplains provide care only to those whose faith is similar to their own (Gatrad et al. 2003). In this article, we will use the term “interfaith spiritual care” in a broad sense, indicating a situation wherein caregiver and patient have different spiritual, religious or non-spiritual, or non-religious worldviews. This implies, for example, an Islamic spiritual caregiver and a Christian patient, but it may also imply a situation wherein one has an explicit religious or spiritual orientation and the other has not, such as a Catholic nurse caring for an agnostic patient.

Although an interfaith approach may be one of the ways to provide spiritual care to patients and clients with diverse spiritual needs, the practice of interfaith spiritual care (ISC) has been contested. For example, Fawcett and Noble (2004) hypothesized that, for Christian nurses, providing spiritual care to patients, who hold very different beliefs from their own, may be challenging, especially with regard to maintaining professional and religious integrity. Others noted the limits of an interfaith approach for chaplains with regard to worship with patients of another faith than their own (Gatrad et al. 2003, 2004) or objected to an interfaith approach because they saw it as an extension of a “Protestant-based” chaplaincy model (Abu-Ras and Laird 2011). Additionally, Ganzevoort et al. (2014) point out that the possibility of providing ISC depends on various factors, one of them being the perspectives on spiritual caregiving within the spiritual tradition of the (spiritual) caregiver. The spiritual care model favored in an Islamic perspective differs for example from the spiritual care model favored in a Buddhist perspective, the first using actions such as reciting the Qur’an and advising patients whether certain practices are acceptable or not, and the second being characterized by practicing meditations as a form of contemplative care. These different spiritual care models may pose challenges to providing ISC.

In summary, spiritual care may often operate in encounters where the caregiver and receiver are from different religious or spiritual background, but little is known about the ways in which ISC meets patients’ diverse, spiritual needs, and there is debate concerning its practice. To date, an overview of what is actually happening in practices of interfaith spiritual care is lacking. Aiming to provide a starting point in finding that knowledge, the objective of this review was to provide an overview of recurring themes in empirical literature on interfaith spiritual care (ISC) in a professional caring relationship.

## Method

A systematic review of empirical studies on ISC was conducted (Higgins et al. 2008).

### Search Strategy

On April 2, 2015, we conducted a search, which was updated on January 18, 2016. We searched in the following databases: PsycINFO, EMBASE, Medline, CINAHL, ATLA Religion database, and Philosopher’s index. The terms “interfaith,” “spiritual,” “care,” as well as synonyms and closely related words were used. These three terms were combined using the Boolean operator AND.

Articles published before 2000 were excluded. Other exclusion criteria were: not written in English, German, French or Dutch, conference abstracts, editorials, book or article reviews or replies, or missing abstract. Empirical studies were included when they described ISC in a professional caring relationship.

**Fig. 1:**
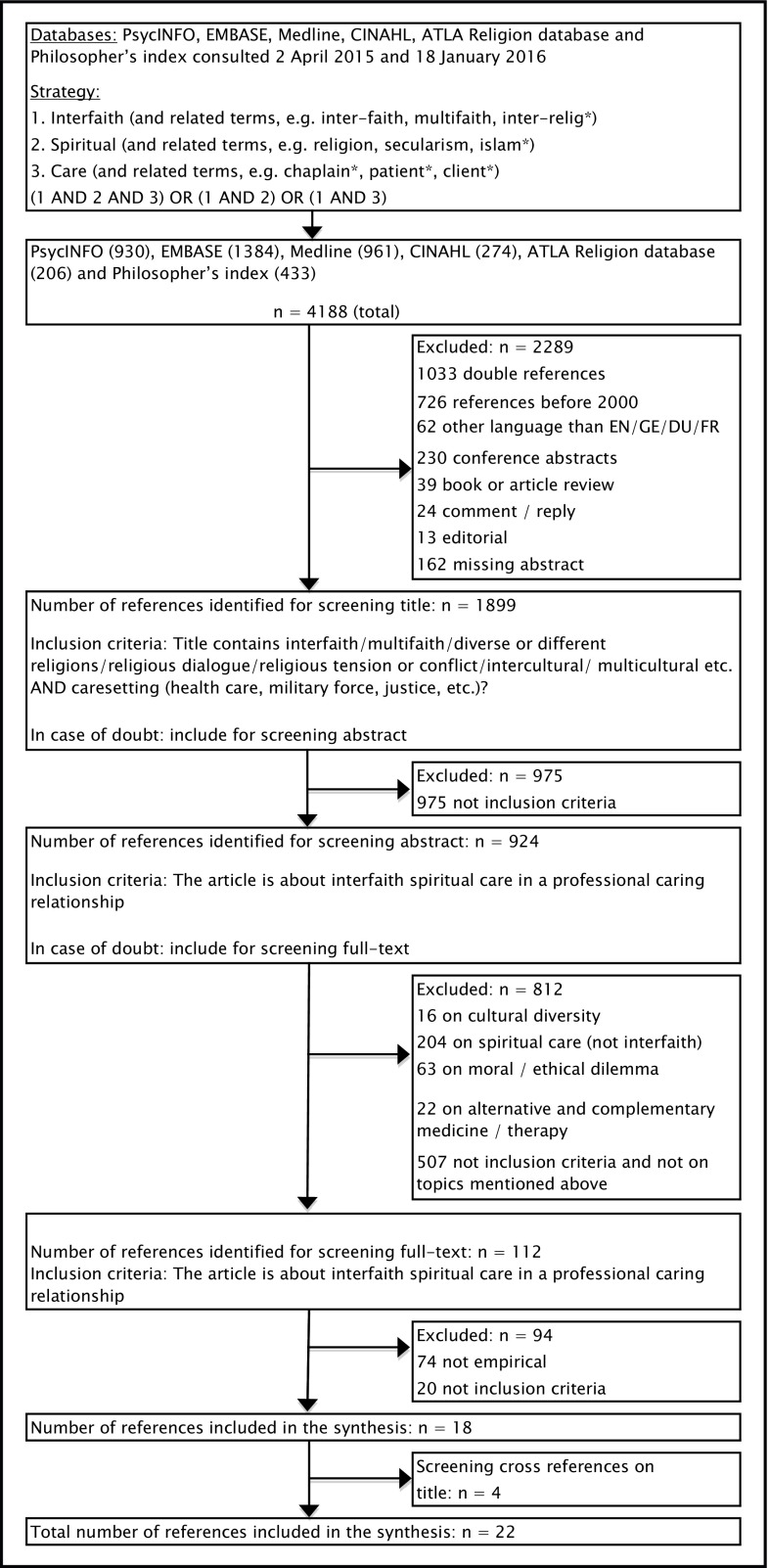
Flow chart

Two researchers screened the titles of the references separately, and then compared and discussed their results, which led to included and excluded references for the next round. In case of doubt, articles were included for the next round. In case of disagreement, a third researcher screened the title and made a final decision. For the title and abstract screening (second round) and the full-text screening (third round), a similar procedure was followed. The cross-references were also screened. For the flow chart, see Fig. 1.

### Data Analysis

Firstly, the characteristics of the included studies were summarized (see Table 1). Secondly, in order to guarantee a minimum of quality of the included studies (Evans 2004), their quality was assessed and a risk of bias was formulated. Since the studies had employed various methodologies, such as in-depth interviews, surveys, and participant observations, different guidelines were used to assess their quality (Tong et al. 2007; Kelley et al. 2003; Leech and Onwuegbuzie 2010). These guidelines provided checklists with items that helped to assess whether the included studies had reported about the items at all (transparency as a first criterion), and if so, what they had reported about these items (validity as a second criterion). Both criteria helped to formulate a risk of bias. More specifically, two researchers formulated a risk of bias independently and, in case of disagreement, discussed their findings until consensus was reached. All included articles were considered to be of sufficient quality, and as a consequence, the results of all studies were used during the next stage.

**Table 1:** Characteristics of the included studies

Author(s) (year)	Objective	Methods	*N*	Participants	Setting	Country	*Q* ^a^	Risk of bias
Abu-Ras (2011)	To examine the roles of Muslim chaplains in health care settings, and in serving Muslim patients in particular	Survey + interviews	56 + 33	Pastoral care directors, chaplains (Muslim and non-Muslim)	Health care	US	+	Not described why using mixed methods was necessary and how the data are integrated
Abu-Ras and Laird (2011)	To examine Muslim and non-Muslim chaplains’ approaches of pastoral care with Muslim patients	Interviews	33	Chaplains (Muslim and non-Muslim)	Hospital	US	+	Poor description of data analysis
Cadge and Sigalow (2013)	To explore chaplains’ two main strategies when working with patients and families with a RS background other than their own	Interviews + pt observ	20	Chaplains (various RS^a^ backgrounds)	Hospital	US	±	Poor description of influence research team on research process; poor description of data collection and analysis
Carey and Davoren (2008)	To explore the interfaith pastoral care provided by Christian health care chaplains to patients and their families of non-Christian religions	Survey + interviews	30	Chaplains (Christian)	Hospital	AU	±	Poor description of qualitative data analysis, small sample for quantitative part
Chui and Cheng (2013)	To explore the experiences of religious workers in Asian penitentiaries	Interviews	17	Prison chaplains, volunteers (Buddhist)	Prison	CN	±	Poor description of influence research team on research process; poor description of data analysis
Ellis and Campbell (2005)	To explore the importance of concordant belief systems in patient–physician spiritual interactions	Interviews	20	Family physicians, (ambulatory) patients	Community health	US	++	None
Galek et al. (2010)	To examine the degree to which chaplains are more likely to pray with patients of their own religious faith	Correlational study	82	Chaplains, students (Christian, Jewish)	Hospital	US	±	Poor description of sample and selection of sample; mainly students
Hodge and Lietz (2014)	To examine the utility of spiritually modified cognitive-behavioral therapy with the treatment of substance abuse	6 focus groups	40	Therapists, clients	Various	US	+	Poor description of influence research team on research process
Kale (2011)	To examine how spiritual care is perceived by recording the lived experiences of palliative care workers at Hospice Africa Uganda	Interviews	15	Various palliative care workers (mainly Christian)	Hospital	UG	±	Poor description of influence research team on research process; poor description of data analysis
Kellems et al. (2010)	To (1) gain information about therapy involving RS issues, (2) examine relationship between similarity of therapist–client RS and therapy process, (3) examine relationship between therapists’ level of personal RS commitment and importance they attach to specific RS goals/interventions, (4) examine the relationship of RS training to self-efficacy for working with RS issues, (5) explore how personal RS impacted therapists’ work with particular clients	Survey	220	University counseling center therapists (various RS backgrounds)	University	US	++	None
Kerley et al. (2009)	To study the narrative of prison chaplains and local religious congregants in order to learn more about the ministry workers responsible for the provision of faith-based prison programs	Interviews	30	Chaplains, religious congregants (Christian)	Prison	US	±	Poor description of data analysis; no code tree
Magaldi-Dopman et al. (2011)	To offer an in-depth, qualitative examination of spiritual/religious/non-religious identity development among psychologists and its impact on their psychotherapy with clients	Interviews	16	Psychologists (various RS backgrounds)	Various	US	++	None
Mayers et al. (2007)	To explore the process of help-seeking and therapy among clients with religious or spiritual beliefs	Interviews	10	Clients (strong RS beliefs; various RS backgrounds)	Therapy	UK	++	None
Pesut and Reimer-Kirkham (2010)	To analyze the negotiation of religious and spiritual plurality in clinical encounters, and the contexts that shape that negotiation	Interviews + pt observ	65	Health care professionals, administrators, patients, family members	Hospital	CA	+	Poor description of the research team and relation of the research team with participants; poor description of data analysis
Pesut et al. (2012)	To examine the contributions of spiritual care providers in Canadian institutional health care contexts	Interviews	21	Spiritual care providers, volunteers (various RS backgrounds)	Hospital	CA	+	Poor description of influence research team on research process; poor description of data analysis
Reimer-Kirkham et al. (2004)	To examine the contexts of intercultural spiritual caregiving	Interviews + focus group	6	Nurses, chaplains	Hospital	CA	+	Small sample; no code tree
Reimer-Kirkham et al. (2012)	To examine the negotiation of religious and spiritual pluralism in health care, with a focus on the themes of “sacred” and “place”	Interviews + pt observ	69	Health care professionals, administrators, patients, family members	Hospital	CA	±	Poor description of influence research team on research process; participant selection and data collection unclear
Sherwood (2000)	To use guided reflection to examine a written caregiving encounter to identify spiritual themes and interpret their influence on nursing practice	5 focus groups	40	Nurses, student nurses	Hospital	US	+	None
Silton et al. (2013)	To obtain basic information from professional chaplains about their use of prayer with patients	1 focus group	8	Chaplains (various RS backgrounds)	Hospital	US	±	Poor description of influence research team on research process; small sample size
Sinclair et al. (2009)	To examine the factors affecting the practice of spiritual care programs or professional chaplains working within an oncology setting	Interviews + pt observ	X	Spiritual caregivers	Cancer center	US	±	Poor description of influence research team on research process; poor description of data collection; no code tree
Taylor et al. (2014)	To describe how the religiosity of Christian nurses motivates their practice and manifests during patient care, especially spiritual care	Interviews	14	Nurses	Hospital	US	++	None
Wesley et al. (2004)	To analyze the roles and educational needs of hospice social workers regarding assessment and intervention in spirituality, religion, and diversity of their patients	Survey	62	Hospice social workers (Various RS backgrounds)	Hospice	US	+	Data collection via membership organization; limited response rate

X, Not applicable; AU, Australia; CN, China; UG, Uganda; RS, Religious/spiritual; pt observ, participant observations; ±, moderate quality; +, good quality; ++, very good quality

^a^Quality was assessed using the following guidelines: Tong et al. (2007) for qualitative studies, Kelley et al. (2003) for quantitative studies, and Leech and Onwuegbuzie (2010) for mixed methods studies

Thirdly, in order to identify recurring themes that emerged in the data, all included articles were inductively analyzed by the first author, using an iterative process of coding and recoding. These preliminary results were discussed in a research group consisting of three researchers, leading to a classification of themes.

## Results

### Characteristics of the Included Articles

Twenty-two articles were included, of which seventeen qualitative (of which three shared the same database), three quantitative, and two mixed method studies. Seventeen focused exclusively on professionals, the majority studying spiritual care providers, and a minority exploring the perspectives of other professionals, like nurses, physicians, psychologists, social workers, and directors. Four (of which two shared the same database) examined both professionals and patients’ or clients’ perspectives and one study focused exclusively on clients’ perspectives. Participants came from a variety of religious or spiritual backgrounds. Eighteen of the twenty-two included studies had been conducted in the USA or Canada, and most studies had been conducted in health care settings, whereas a few had been conducted in other settings, like prison or university. The majority of the articles were of sufficient or high quality, whereas some of them were of very high quality. For details on the included studies, see Table 1.

### Providing Interfaith Spiritual Care: Normativity and Capacity

The included articles used diverse terms when discussing caregiving to patients of various faiths, such as “concordant and discordant spiritual orientations in physician-patient spiritual discussion” (Ellis and Campbell 2005), “multi-faith chaplaincy,” “general prayer” (Pesut et al. 2012), “universal and non-denominational” prayer (Kale 2011) and “interfaith” and a “faith-specific chaplaincy approach” (Abu-Ras and Laird 2011). Not all of those terms were clearly defined.

Nevertheless, recurring themes were identified in the thematic analysis of the included studies, leading to two core categories that were different, yet related: normativity and capacity. Normativity regards an answer to the question: do I want to provide ISC or not, and for which reasons? Capacity implies an answer to the question: is it possible to provide ISC or not, and for which reasons? For a schematic overview of the results, see Tables 2 and 3.

**Table 2:** Thematic analysis: normativity

Normativity: Reasons for (not) wanting to provide ISC	Universalist approach	Example universalist approach	Other references	Particularist approach	Example particularist approach	Other references
Identity characterized by…	Openness to diverse perspectives	Several ministry workers enjoyed interacting with inmates from different faiths, because they feel it is intellectually and religiously rewarding to learn about different faith traditions (Kerley et al. 2009)	Cadge and Sigalow (2013), Ellis and Campbell (2005)	Focus on specific faith	“Muslims should be treated as individuals with specific needs” (Abu-Ras and Laird 2011)	Chui and Cheng (2013)
	Emphasizing importance of a spiritual care service for everyone	Faith-based service creates restrictions for people not identifying with that tradition (Sinclair et al. 2009)	Reimer-Kirkham et al. (2004), Silton et al. (2013)	Emphasizing risks of a spiritual care service for everyone	Risk of faith being challenged by learning about “different ways to God” (Carey and Davoren 2008)	Hodge and Lietz (2014), Ellis and Campbell (2005), Magaldi-Dopman et al. (2011), Reimer-Kirkham et al. (2004), Kale (2011), Sinclair et al. (2009)
Relationships characterized by…	Spiritual connections across faiths	By discussing spiritual/religious topics in psychotherapy—even as an atheist psychologist—“a spiritual connection [was] formed, a feeling of the transcendent, or a ‘religious moment’ where they felt the presence of something larger than themselves” (Magaldi-Dopman et al. 2011)	Kellems et al. (2010), Pesut and Reimer-Kirkham (2010), Pesut et al. (2012), Reimer-Kirkham et al. (2004), Silton et al. (2013)	Spiritual connection with same faith	Sharing the same belief system and cultural background facilitates spiritual interaction (Ellis and Campbell 2005)	Hodge and Lietz (2014), Silton et al. (2013), Sinclair et al. (2009), Kellems et al. (2010), Kerley et al. (2009), Magaldi-Dopman et al. (2011)
	Patients’ preferences that do not imply a specific faith	Most patients speak of wanting “kindness, respect, humor, and friendship” when asked about spiritual care by a caregiver (Pesut and Reimer-Kirkham 2010)	Abu-Ras and Laird (2011), Mayers et al. (2007)	Patients’ preferences that imply a specific faith	Patients having strong R/S beliefs felt dilemmas about contacting secular health care services, because of fear it might be seen by God, others and themselves as a rejection of God’s healing, or they thought it would be impossible to discuss spiritual topics in therapy, or because of fear of “conversion” by “antireligious beliefs” (Mayers et al. 2007)	
				Fear of imposing own beliefs on client or being viewed as proselytizing	Both religious and secular therapists note the risk of “imposing their own personal beliefs,” thereby not respecting the clients’ autonomy (Hodge and Lietz 2014)	Kellems et al. (2010), Silton et al. (2013), Sinclair et al. (2009), Taylor et al. (2014), Ellis and Campbell (2005)
Actions characterized by…	Prayer across faiths	Prayer was identified as an important tool “that could be shared with patients of all faiths, as it can be universal and non-denominational” (Kale 2011)	Silton et al. (2013), Pesut et al. (2012)	Prayer and rituals with same faith	Some patients note they prefer a chaplain reciting prayers from their own tradition and discordant prayer might be a source of tension (Silton et al. 2013)	Abu-Ras and Laird (2011), Abu-Ras (2011), Galek et al. (2010)

**Table 3:** Thematic analysis: capacity

Capacity needed for providing ISC	Specification	Example	Other references
Competence	Strategies	Being able to neutralize and code-switch (Cadge and Sigalow 2013); taking a “patient-centered viewpoint” (Ellis and Campbell 2005); using “non-religious language” when engaging in conversation with a non-religious patient (Taylor et al. 2014)	Pesut and Reimer-Kirkham (2010), Pesut et al. (2012), Abu-Ras and Laird (2011), Kerley et al. (2009), Carey and Davoren (2008), Mayers et al. (2007)
	Knowledge/recognition	Care providers, when coming from a different religious/cultural background than their patients, might not recognize the importance of religion/religious practices for patients (Pesut and Reimer-Kirkham 2010)	Abu-Ras (2011), Silton et al. (2013), Ellis and Campbell (2005), Wesley et al. (2004), Carey and Davoren (2008), Magaldi-Dopman et al. (2011)
Context	Individual level—possibilities	Patients may experience the visit of someone of another tradition/religion as a privilege or honor (Silton et al. 2013)	None
	Individual level—restraints	According to a Catholic chaplain, when visiting Catholic patients, “their expectations, and all the traditions get in the way” (Silton et al. 2013)	Cadge and Sigalow (2013), Silton et al. (2013), Kale (2011), Pesut and Reimer-Kirkham (2010), Reimer-Kirkham et al. (2004), Abu-Ras and Laird (2011)
	Institutional level—possibilities	A faith-based spiritual care service received no institutional funding, whereas for those services who used a non-denominational/multifaith approach, it was more likely to receive monetary support by the institution (Sinclair et al. 2009)	Ellis and Campbell (2005)
	Institutional level—restraints	None	None

#### Normativity

A difference was found between a universalist approach, favoring ISC, and a particularist approach, mainly opposing ISC. They implied a different (normative) view on identities, relationships, and actions, by answering the following questions in different ways: who do I want to be (identities)? What kind of professional caring relationships do I want to establish (relationships)? And what do I want to do (actions)? A universalist approach implied an identity that was characterized by an open attitude, a caring relationship that was described in terms of spiritual connection, and it implied actions, particularly prayer, which transcended a specific religion. A particularist approach, on the other hand, meant an identity characterized by a visible connection to a particular religion/spirituality, and a caring relationship characterized by the same spiritual background. A particularist approach also included actions that aimed at connecting caregivers and patients with the same spiritual background. Both approaches will be elucidated now.

##### Normativity: A universalist approach

 A universalist approach toward ISC meant in the first place that participants wanted to be open toward other spiritualities because an open attitude facilitated discussions on spiritual topics (Cadge and Sigalow 2013; Ellis and Campbell 2005).

Secondly, participants in several studies described their interfaith caring relationship itself in spiritual terms, like a “wonderful connection” (Silton et al. 2013), “a ‘religious moment’ where they felt the presence of something larger than themselves” (Magaldi-Dopman et al. 2011), and a “bond” instead of a “barrier” (Reimer-Kirkham et al. 2004). In addition, in a large survey among university counseling center therapists, the perceived similarity between therapists’ and clients’ religious or spiritual values was not associated with the strength of the therapeutic relationship (Kellems et al. 2010), and when Pesut and Reimer-Kirkham (2010) asked patients (*n* = 16) how they felt about spiritual care by a caregiver, most patients spoke about wanting “kindness, respect, humor, and friendship,” not mentioning a particular religious background of their caregiver. Most non-Muslim chaplains in another study (Abu-Ras and Laird 2011) suggested that Muslim patients often do not need an imam (or a substitute in their absence) because, for example, they are secularized or their needs will be met another way.

Thirdly, a universalist approach implied universal actions, and prayer was mentioned frequently. Participants in two studies noted the beauty of multifaith or “general” prayer (Pesut et al. 2012; Silton et al. 2013), and a study conducted in Uganda (Kale 2011) found that interfaith “prayer was considered a very important tool that could be shared with patients of all faiths, as it can be universal and non-denominational.”

##### Normativity: a particularist approach

 Other findings supported a particularist approach, mainly favoring faith-based spiritual care. For example, several chaplains in one study (Abu-Ras and Laird 2011) noted the importance of treating patients as individuals with specific needs, who might best be cared for by someone of the same religious background. Sinclair et al. (2009), in addition, found that a multifaith spiritual care model was perceived by some chaplains as a “diluted form of spiritual care,” suggesting they may be afraid of losing the particularity of their own (spiritual) identity. According to more than half of the included thirty Christian chaplains in another study, learning about “different ways to God” challenged their own faith (Carey and Davoren 2008).

A particularist approach was also supported by studies that reported difficulties in relationships and interactions when patients and caregivers had different beliefs (Ellis and Campbell 2005; Magaldi-Dopman et al. 2011; Reimer-Kirkham et al. 2004; Taylor et al. 2014; Kerley et al. 2009). For example, some caregivers were hesitant to provide ISC because they feared being viewed as proselytizing or imposing their own beliefs on patients, thereby not respecting patients’ autonomy (Silton et al. 2013; Hodge and Lietz 2014). Sharing the same belief system, in addition, facilitated spiritual interaction (Ellis and Campbell 2005), brought confidence and comfort (Ellis and Campbell 2005; Silton et al. 2013), served as a “point of unity” (Sinclair et al. 2009), and facilitated social support (Hodge and Lietz 2014). Silton et al. (2013) provided another example favoring a particularist approach: they found that discordant prayer—praying with someone of a different faith—might be a source of tension, and patients in their study preferred a chaplain reciting prayers from their own tradition.

In terms of actions, a particularist approach aimed to connect caregivers and patients with the same spiritual background. Abu-Ras and Laird (2011) reported, for instance, how a chaplain referred patients requesting a religious ritual to a chaplain with the same spiritual background. In addition, chaplains in another study were more likely to pray with people from the same religion than with patients who adhered to a different religion (Galek et al. 2010).

#### Capacity

The capacity to provide ISC included health care professionals’ competences, and the possibilities and restraints of the context in which ISC was provided. Health care professionals needed the capacity to create a third space, in-between two spiritual worlds/discourses, and knowledge of other spiritualities was required. Contextual restraints and possibilities, included, among other things, the name “chaplain” and her/his denomination, and health care institutions favoring ISC instead of faith-based approaches. The capacity to provide ISC will be described in detail now.

##### Capacity: competence

 Some of the included studies identified strategies caregivers used to provide ISC, and they seemed to assume the capacity to create a third, relational space, in-between two discourses or worlds. Cadge and Sigalow (2013), for instance, noted the ability of “neutralizing” (emphasizing commonalities) and “code-switching” (moving between different religious languages, symbols, and practices). The notion of focusing on similarities between various beliefs by volunteers and chaplains in the study of Kerley et al. (2009) also reflected their ability to “neutralize.” In a similar vein, Pesut and Reimer-Kirkham (2010) spoke about eliciting patients’ meaning systems “as a means to transcend difference and create safe sacred spaces” and, in another study by Pesut et al. (2012), about spiritual care providers creating “sacred, inclusive spaces and language.” Within this relational space, the importance of focusing on the patients’ perspective was mentioned frequently in the included studies. Ellis and Campbell (2005) noted for instance that, according to physicians as well as patients, taking a “patient-centered viewpoint” is one of the approaches physicians need when engaging in conversation with patients holding different beliefs. Likewise, Taylor et al. (2014) mentioned the use of “non-religious language” when engaging, as a Christian nurse, in conversation with a non-religious patient. Mayers et al. (2007) reported that “acceptance, respect, understanding and then a willingness to work with, and not against, the participant’s way of viewing the problem and ideas about a solution enhanced the development of a sound therapeutic relationship.”

In addition to this relational space, several studies suggested that knowledge of other spiritualities reinforced the capacity to provide ISC, and a lack of this knowledge hindered ISC (Wesley et al. 2004; Carey and Davoren 2008). Others found that health care professionals, when coming from a different religious or cultural background than their patients, may not have the capacity to recognize the importance of spirituality for patients or diagnose their spiritual needs (Pesut and Reimer-Kirkham 2010; Abu-Ras 2011). The study of Magaldi-Dopman et al. (2011) forms an exception: they found that atheist and agnostic psychologists were more likely than psychologists with a religious or spiritual background to “pay close attention to religious issues in psychotherapy because they were afraid to overlook this area because of their own beliefs.” Thus, whereas some authors reported the lack of knowledge and recognition of various spiritualities created barriers to ISC, fear of not being able to recognize the role of certain beliefs may also create an incentive to attend to those topics.

##### Capacity: context

 Besides the individual competences of health care professionals, there were also contextual possibilities and restraints that shaped the capacity for providing ISC. Most of these were reported in chaplaincy studies. At the individual level, several included studies reported impediments to providing ISC, like language differences (Kale 2011; Pesut and Reimer-Kirkham 2010; Reimer-Kirkham et al. 2004), gender issues, such as rejection when visiting someone of the opposite sex (Pesut and Reimer-Kirkham 2010; Abu-Ras and Laird 2011), and politics (Abu-Ras and Laird 2011). Another restraint was the name “chaplain” and the denomination of the chaplain. A Jewish and a Muslim chaplain, in one study, for example mentioned that the Christian connotation of the term “chaplaincy” sometimes made Jewish or Muslim patients reject spiritual care (Cadge and Sigalow 2013). In addition, Silton et al. (2013) reported that there seemed to be confusion concerning the name “chaplain,” especially for Muslim and Jewish patients, and that Jewish patients sometimes asked for a “rabbi” instead of a (multifaith) chaplain because they were unfamiliar with the term “chaplaincy.” However, they also described that the denomination of the chaplain might offer possibilities. A Catholic chaplain in their study explained that, when visiting Jewish patients, these patients experienced this as a privilege or honor, while when visiting Catholic patients, “their expectations, and all the traditions get in the way.”

At the institutional level, only possibilities for providing ISC were mentioned, mainly reported by Sinclair et al. (2009). They noted, for instance, that a spiritual care service that “understood its mandate as tending to the spiritual needs of the diverse clientele of its institution was more likely to be recognized as a formal service by health care staff (…).” At one of the sites, visited by the same researchers, the faith-based spiritual care service received no institutional funding because it was “identified by administration as a representative of their faith-based institution rather than a professional attending to an important aspect of universal human health.” Furthermore, spiritual care services that used a non-denominational and multifaith approach were more likely to receive institutional funding.

## Discussion

This review provides an overview of twenty-two empirical studies on interfaith spiritual care (ISC) in a professional caring relationship, suggesting that there are (at least) two categories involved in ISC: normativity and capacity. The first category—normativity—means the reasons for (not) wanting to provide ISC, consisting of a universalist and particularist approach. The second category—capacity—consists of reasons for (not) being able to provide ISC, which included competences needed to provide ISC and contextual possibilities and restraints.

The first category, normativity, relates to the legitimacy of ISC. Firstly, from the perspective of certain spiritual traditions such as Christian ones, it is important to care for everyone (Schipani and Bueckert 2009), which may (partly) support a universalist approach. In addition, from an organizational or institutional perspective ISC appears to be legitimate, as it fits the aim of national health services to care for patients regardless of their religious background (National Health Service 2015) and because it is more likely to receive funding (Sinclair et al. 2009). ISC may be legitimate from the perspective of patients as well. Some of the results in this review study suggest that, according to patients, the caregivers’ religious orientation does not play a major role in spiritual caregiving, although it may be important for a minority group (Abu-Ras and Laird 2011; Pesut and Reimer-Kirkham 2010). However, no definitive conclusions can be drawn from the limited research conducted thus far, and future research should further explore the legitimacy of ISC.

The second category—capacity—highlights important competences required to provide ISC, for example code-switching and neutralizing (Cadge and Sigalow 2013). However, we should ask how reasonable it is to expect from (spiritual) caregivers that they have in-depth knowledge of all spiritual traditions. Moreover, this review suggests that there are contextual restraints that hinder ISC, and therefore training of competences may only be one condition for providing good ISC. Thirdly, the included studies have mainly described prayer and (verbal) communication on spiritual issues, which reflects a view on spiritual care that prevails in Protestant Christianity but is less central to other spiritual traditions such as Islam (Abu-Ras and Laird 2011), Buddhism, or neo-paganism. A broader concept of spiritual care should include the performance of rituals, meditations, education, and advice-giving (Ganzevoort et al. 2014). Further research on these various dimensions of ISC is necessary.

Although this review demonstrates that some initial research has been conducted on the topic of ISC, there still seem to be several gaps in the literature on this issue, as illustrated by the limitations of the included studies in this review. First, the number of empirical studies identified is small (twenty-two), and the data gathered in these studies are limited because of small sample sizes. As a consequence, we should be cautious in generalizing the findings of these studies. In qualitative meta-analysis, however, the aim is not statistical generalization but theory-building through transferability. The insights thus may be considered to be transferable rather than generalizable.

Secondly, the majority of the included studies were conducted in the USA and Canada, which is in line with the findings of a recent review study on chaplaincy research at large (Pesut et al. 2016). Since the religious and spiritual landscape in North America differs from other Western societies, for instance Europe (Berger et al. 2008), and even more from non-Western societies, future research should also be conducted in other cultural contexts.

Since most of the included studies used self-report approaches to investigate ISC, the way ISC encounters actually take place have hardly been explored. Moreover, the included articles in this review study mainly focused on professional caregivers’ perceptions, and only five of them have examined clients’ or patients’ perspectives. This limitation necessitates the exploration of patient perspectives on ISC in future studies.

This study identified various terms that were used describing approaches to care for patients of diverse spiritual or religious backgrounds. Since this terminology varies widely and sometimes lacks a clear definition and conceptual framework, not only more research has to be done to investigate practices of ISC, but also to explore its theoretical assumptions.

One of the strengths of this systematic review is that it provides insight into some key issues in ISC. In addition, it shows the current state of affairs with respect to research on ISC by providing an overview of what is already investigated (and what is not) in empirical research on this topic.

## Conclusion

The knowledge gained through this systematic review helps to understand some key issues in interfaith spiritual care (ISC) within a landscape characterized by a diversity of spiritual needs. It provides an overview of what is known regarding ISC and identifies gaps in the literature. It indicates, for instance, that future studies should investigate what ISC encounters actually look like in practice, and that future studies should explore patients’ perspectives on ISC, in order to learn how ISC contributes to patients’ spiritual wellbeing. Our hope is that these future studies, together with the results presented in this review study, will contribute to good spiritual care that attunes to patients and their family members with a diversity of spiritual needs and backgrounds.
